# Otologic Surgery Risk Prediction: Risk Analysis Index‐Administrative Versus Modified Frailty Index‐5

**DOI:** 10.1002/lary.70539

**Published:** 2026-04-03

**Authors:** Akshay Warrier, Nicole Kim, Ryan Bartholomew, Liliya Benchetrit, Daniel J. Lee

**Affiliations:** ^1^ Department of Otolaryngology—Head and Neck Surgery Rutgers New Jersey Medical School Newark New Jersey USA; ^2^ Department of Otolaryngology—Head and Neck Surgery, Massachusetts Eye and Ear Harvard Medical School Boston Massachusetts USA; ^3^ Department of Otolaryngology—Head and Neck Surgery Harvard Medical School Boston Massachusetts USA

**Keywords:** frailty, modified frailty index, otologic surgery, postoperative outcomes, risk analysis index

## Abstract

**Objectives:**

Frailty indices, including the modified frailty index (mFI‐5) and the risk analysis index‐administrative (RAI‐A), are increasingly used to stratify surgical risk; however, their comparative utility in otology/neurotology remains understudied. This study compares the predictive performance of RAI‐A versus mFI‐5 for postoperative outcomes in a large otologic and lateral skull base cohort.

**Methods:**

A retrospective analysis of 2862 otologic surgery patients was performed. Primary outcomes included 30‐day mortality, Clavien–Dindo (CD) complications, surgical site infection (SSI), nonhome discharge, and extended length of stay (eLOS). Frailty was assessed using validated indices, and predictive performance was evaluated using multivariate logistic regression and ROC curves.

**Results:**

Across the full cohort, RAI‐A demonstrated superior discrimination (C‐statistic) compared to mFI‐5 for mortality (0.809 vs. 0.722, *p* = 0.021), CD II complications (0.793 vs. 0.788, *p* = 0.048), and SSI (0.721 vs. 0.712, *p* = 0.047). On multivariable analysis, RAI‐A was significantly associated with CD II (OR 7.545; CI 2.367–24.048), CD IIIb (OR 17.925; CI 1.641–195.753), and eLOS (OR 40.623; CI 4.966–336.395). In the skull base subset, RAI‐A was associated with higher odds of mortality (OR 14.47, 95% CI 3.02–69.31), serious complications (CD IV; OR 3.56, 95% CI 1.36–9.31), and nonhome discharge (OR 18.20, 95% CI 6.35–52.17).

**Conclusion:**

RAI‐A outperforms mFI‐5 in predicting postoperative outcomes in otologic surgery, with similar findings for high‐risk lateral skull base cases. These findings support incorporating RAI‐based frailty assessment into preoperative planning to improve risk stratification and patient counseling.

**Level of Evidence:**

3.

## Graphical abstract

In a national cohort of 2862 patients undergoing otologic surgery, the risk analysis index‐administrative (RAI‐A) outperformed the modified frailty index‐5 (mFI‐5) in predicting key 30‐day postoperative outcomes, including mortality, complications, surgical site infection, extended length of stay, and nonhome discharge. RAI‐A showed stronger discrimination overall and retained independent associations with multiple adverse outcomes on multivariable analysis, with similar or greater utility in the higher‐risk lateral skull base subgroup. These findings support RAI‐A as a more clinically useful frailty measure for preoperative risk stratification and perioperative counseling in otologic surgery.

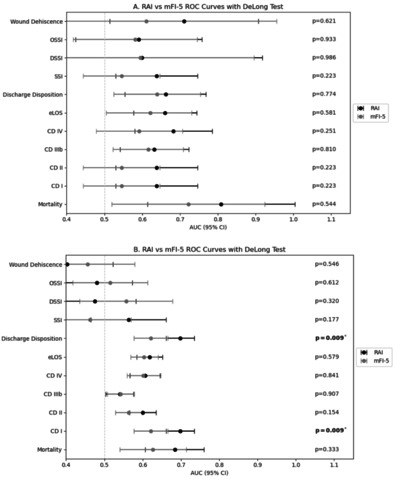

## 1 Introduction

Although otologic surgery is often elective and associated with low mortality, 5%–10% of otologic surgeries have postoperative complications leading to hospital readmission or unplanned revisits [1, 2]. Therefore, identifying patients at elevated risk could improve perioperative planning and help avoid procedures in individuals who are poor surgical candidates. Traditionally, age and comorbidity burden have been used as proxies for patients' overall health status [3, 4]. However, these variables may not fully capture a patient's ability to withstand physiologic stressors such as surgery. Thus, the concept of frailty has emerged as a framework to capture a patient's physiologic reserve to stress; it has been shown to be superior to age alone in predicting postoperative complications in various surgical subspecialties [5, 6, 7, 8].

Multiple screening tools have been developed and validated to quantify frailty in surgical populations [9, 10]. Among these, the 5‐item modified frailty index (mFI‐5), derived from the American College of Surgeons National Surgical Quality Improvement Program (ACS‐NSQIP), operationalizes frailty using five binary variables to produce a score between 0 and 5: functional status, diabetes, history of COPD, history of CHF, and hypertension requiring medication [11]. The risk analysis index (RAI) is another frailty assessment tool that has been validated for use in surgical populations [10]. The RAI is offered as both a clinical questionnaire for prospective use (RAI‐C) and can be calculated retrospectively (RAI‐A). Compared to the mFI‐5, the RAI generates a broader, granular range of scores from 0 to 81 by incorporating additional variables such as a patient's cognition, activities of daily living, and place of residence [10].

In otology, prior studies have found that mFI‐5 outperforms age alone in predicting outcomes such as readmission and prolonged length of stay [8, 12, 13, 14]. However, the comparative performance of frailty indices in otologic and lateral skull base surgery remains poorly defined. Accordingly, this study compares the predictive utility of the mFI‐5 and the RAI‐A for postoperative complications following otologic and lateral skull base procedures. We hypothesized that the multidimensional RAI‐A would outperform the mFI‐5 in predicting postoperative complications, mortality, prolonged hospitalization, and discharge disposition.

## 2 Materials and Methods

### 2.1 Data Source

This was a retrospective cohort study utilizing the ACS‐NSQIP database from 2005 to 2020, a nationally validated, multi‐institutional program that collects de‐identified surgical data from participating hospitals in the United States, Canada, and international sites. Adult patients undergoing otologic or neurotologic surgery were identified using current procedural terminology (CPT) codes selected a priori based on clinical relevance (Table S1). A subset of lateral skull base procedures was examined as a predefined high‐risk cohort but should be interpreted as exploratory given the small sample size and limited event counts. Patients were excluded if frailty elements or 30‐day outcomes were not recorded. The ACS NSQIP includes both inpatient and outpatient procedures performed in operating room and procedural suite settings. To capture the full procedural spectrum of otologic surgery, we included all otologic and neurotologic CPT codes meeting inclusion criteria. A sensitivity analysis excluding low‐acuity, office‐based procedures (e.g., cerumen removal, auricular hematoma evacuation, simple myringotomy) was performed to confirm robustness.

### 2.2 Frailty

Frailty was assessed using two validated indices. The mFI‐5 was calculated in accordance with standard ACS‐NSQIP methodology using five comorbidity and functional status variables to generate a frailty score ranging from 0 to 5 [9]. The risk analysis index‐administrative (RAI‐A), a retrospectively calculated instrument distinct from RAI‐clinical (RAI‐C), was calculated from NSQIP variables pertaining to age, comorbidity burden, functional independence, weight loss, dyspnea, and residential status, producing a continuous score ranging from 0 to 81 (Table 1) [10].

**TABLE 1:** Components of frailty indices and associated scoring in otologic surgery.

Modified frailty index‐5 (mFI‐5)	Risk analysis index‐administrative (RAI‐A)
*Comorbidities*	*Demographics*
Diabetes mellitus (+1)	Age
Hypertension (+1)	Sex (female = 0, male = +5)
Dependent functional status (+1)	*Comorbidities*
Chronic obstructive pulmonary disease or pneumonia (+1)	Cancer diagnosis excluding melanoma (range 13–20)
Congestive heart failure (+1)	Renal failure or dialysis (+6)
	Congestive heart failure (+4)
	Shortness of breath at rest or minimal activity (+8)
	Residence other than independent living (+8)
	*Functional status*
	Unintentional weight loss of 4.5 kg over 3 months (+5)
	Poor appetite (+4)
	Activities of daily living (ADL) defined as functional status
	Mobility (0–4)
	b Eating (0–4)
	c Toilet use (0–4)
	d Hygiene (0–4)

### 2.3 Outcomes

Primary endpoints included 30‐day mortality, Clavien–Dindo (CD) complications, extended length of stay (eLOS; defined as length of stay greater than or equal to the 75th percentile of the cohort), and nonhome discharge (NHD) [15]. Secondary outcomes included unplanned readmission, reoperation, superficial surgical site infection (SSI), deep SSI (DSSI), and organ‐space SSI (OSSI) within 30 days following the index procedure.

### 2.4 Statistical Analyses

Descriptive statistics were compared using Pearson *χ* ^2^ or Fisher's exact testing for categorical variables and independent sample *t*‐testing or Mann–Whitney *U* testing for continuous variables, as appropriate. Multivariable logistic regression models were constructed to evaluate the association between each categorical frailty measure (with “robust” as reference) and postoperative outcomes while adjusting for potential confounders including age, sex, body mass index, smoking status, American Society of Anesthesiologists (ASA) class, and operative time. To avoid collinearity, the indices were modeled separately, and age was excluded from the RAI‐A model but retained in the mFI‐5 model. Predictive performance and model discrimination were assessed using unadjusted receiver operating characteristic curve analysis with comparison of area under the curve values through the DeLong method. Lateral skull base procedures represent a distinct clinical subset characterized by longer operative times, greater physiologic stress, and higher risk of neurologic and medical complications, warranting separate evaluation. A sensitivity analysis was conducted within the lateral skull base subgroup to assess the performance of frailty indices in a higher‐risk surgical population. All statistical analyses were performed using IBM SPSS Statistics version 29 (IBM Corp., Armonk, NY). Statistical significance was defined as a two‐sided *p* value less than 0.05.

Because this study utilized de‐identified, publicly available NSQIP data, IRB approval was not required.

## 3 Results

### 3.1 Study Population Characteristics

A total of 2862 patients undergoing otologic surgery were included. The mean age was 47.4 ± 17.4 years, and sex distribution was balanced (52.2% male). The majority of patients were White (68.8%), followed by Hispanic (8.7%), Black (7.2%), Asian (4.5%), and other racial groups (11.0%). Baseline demographic characteristics and postoperative outcomes are summarized in Table 2. Hypertension, diabetes, and COPD were significantly more common among frail patients according to both indices (Table 2).

**TABLE 2:** Demographic, clinical, and perioperative characteristics of adult otology patients characterized by frailty screening tool (risk analysis index and modified frailty index‐5, *n* = 2862).

Category		RAI‐A categories				mFI‐5 categories			
	Totals *n* = 2862	Robust, *n* = 2257	Prefrail, *n* = 547	Frail, *n* = 61	Severely frail, *n* = 5	Robust, *n* = 1878	Prefrail, *n* = 701	Frail, *n* = 261	Severely frail, *n* = 29
*Demographics*									
Male, *n* (%)	1494 (52.2%)	1307 (57.91%)	177 (32.36%)	8 (13.11%)	2 (40.00%)	1010 (53.78%)	351 (50.00%)	123 (47.13%)	10 (34.48%)
Female, *n* (%)	1376 (48.1%)	950 (42.09%)	370 (67.64%)	53 (86.89%)	3 (60.00%)	868 (46.22%)	351 (50.00%)	138 (52.87%)	19 (65.52%)
Race, *n* (%)	1970 (68.8%)	1506 (66.73%)	408 (74.59%)	52 (85.25%)	4 (80.00%)	1273 (67.78%)	504 (71.79%)	174 (66.67%)	19 (65.52%)
White, *n* (%)	205 (7.2%)	170 (7.53%)	35 (6.40%)	0 (0.00%)	0 (0.00%)	104 (5.54%)	75 (10.68%)	23 (8.81%)	3 (10.34%)
Black, *n* (%)	129 (4.5%)	104 (4.61%)	24 (4.39%)	0 (0.00%)	1 (20.00%)	94 (5.01%)	22 (3.13%)	12 (4.60%)	1 (3.45%)
Asian, *n* (%)	250 (8.7%)	208 (9.22%)	37 (6.76%)	5 (8.20%)	0 (0.00%)	168 (8.95%)	46 (6.55%)	31 (11.88%)	5 (17.24%)
Hispanic, *n* (%)	316 (11.0%)	269 (11.92%)	43 (7.86%)	4 (6.56%)	0 (0.00%)	239 (12.73%)	55 (7.83%)	21 (8.05%)	1 (3.45%)
Other^a^, *n* (%)	1494 (52.2%)	1307 (57.91%)	177 (32.36%)	8 (13.11%)	2 (40.00%)	1010 (53.78%)	351 (50.00%)	123 (47.13%)	10 (34.48%)
*Preoperative clinical status*									
Diabetes, *n* (%)	319 (11.1%)	197 (8.7%)	106 (19.4%)	14 (23.0%)	2 (40%)	0 (0%)	71 (10.1%)	221 (84.7%)	27 (93.1%)
Hypertension, *n* (%)	859 (30.0%)	484 (21.44%)	332 (60.69%)	38 (62.30%)	5 (100.00%)	0 (0.00%)	572 (81.48%)	258 (98.85%)	29 (100.00%)
CHF, *n* (%)	7 (0.2%)	0 (0.00%)	5 (0.91%)	1 (1.64%)	1 (20.00%)	0 (0.00%)	0 (0.00%)	2 (0.77%)	5 (17.24%)
COPD, *n* (%)	81 (2.8%)	40 (1.77%)	34 (6.22%)	7 (11.48%)	0 (0.00%)	0 (0.00%)	32 (4.56%)	29 (11.11%)	20 (68.97%)
Dyspnea, *n* (%)	114 (4.0%)	44 (1.95%)	51 (9.32%)	17 (27.87%)	2 (40.00%)	34 (1.81%)	45 (6.41%)	29 (11.11%)	6 (20.69%)
Renal failure, *n* (%)	2 (0.1%)	2 (0.09%)	0 (0.00%)	0 (0.00%)	0 (0.00%)	0 (0.00%)	2 (0.28%)	0 (0.00%)	0 (0.00%)
Dialysis, *n* (%)	10 (0.3%)	7 (0.31%)	2 (0.37%)	1 (1.64%)	0 (0.00%)	1 (0.05%)	5 (0.71%)	3 (1.15%)	1 (3.45%)
Disseminated cancer, *n* (%)	29 (1.0%)	2 (0.09%)	1 (0.18%)	24 (39.34%)	2 (40.00%)	15 (0.80%)	10 (1.42%)	3 (1.15%)	1 (3.45%)
Weight loss, *n* (%)	15 (0.5%)	3 (0.13%)	9 (1.65%)	2 (3.28%)	1 (20.00%)	9 (0.48%)	1 (0.14%)	4 (1.53%)	1 (3.45%)
Nonhome origin, *n* (%)	41 (1.4%)	27 (1.20%)	10 (1.83%)	3 (4.92%)	1 (20.00%)	23 (1.22%)	10 (1.42%)	8 (3.07%)	0 (0.00%)
Functional status, *n* (%)	2811 (98.2%)	2232 (98.89%)	526 (96.16%)	53 (86.89%)	0 (0.00%)	1871 (99.63%)	673 (95.87%)	247 (94.64%)	20 (68.97%)
Independent, *n* (%)	41 (1.4%)	15 (0.66%)	16 (2.93%)	6 (9.84%)	4 (80.00%)	0 (0.00%)	22 (3.13%)	11 (4.21%)	8 (27.59%)
Partial dependence, *n* (%)	7 (0.2%)	1 (0.04%)	4 (0.73%)	1 (1.64%)	1 (20.00%)	0 (0.00%)	5 (0.71%)	1 (0.38%)	1 (3.45%)
Total dependence, *n* (%)	1 (0.0%)	2089 (92.56%)	491 (89.76%)	57 (93.44%)	3 (60.00%)	1739 (92.60%)	638 (90.88%)	238 (91.19%)	25 (86.21%)
Elective surgery status, *n* (%)	2640 (92.2%)	484 (21.44%)	332 (60.69%)	38 (62.30%)	5 (100.00%)	0 (0.00%)	572 (81.48%)	258 (98.85%)	29 (100.00%)
*Postoperative outcomes*									
Any wound complication, *n* (%)	53 (1.9%)	30 (1.33%)	18 (3.29%)	5 (8.20%)	0 (0.00%)	28 (1.49%)	16 (2.28%)	8 (3.07%)	1 (3.45%)
SSI, *n* (%)	33 (1.2%)	17 (0.75%)	11 (2.01%)	5 (8.20%)	0 (0.00%)	19 (1.01%)	9 (1.28%)	4 (1.53%)	1 (3.45%)
DSSI, *n* (%)	5 (0.2%)	3 (0.13%)	2 (0.37%)	0 (0.00%)	0 (0.00%)	2 (0.11%)	2 (0.28%)	1 (0.38%)	0 (0.00%)
OSSI, *n* (%)	12 (0.4%)	8 (0.35%)	4 (0.73%)	0 (0.00%)	0 (0.00%)	6 (0.32%)	4 (0.57%)	2 (0.77%)	0 (0.00%)
Dehisc., *n* (%)	4 (0.1%)	2 (0.09%)	2 (0.37%)	0 (0.00%)	0 (0.00%)	1 (0.05%)	2 (0.28%)	1 (0.38%)	0 (0.00%)
Clavien–Dindo II, *n* (%)	49 (1.7%)	22 (0.97%)	17 (3.11%)	9 (14.75%)	1 (20.00%)	18 (0.96%)	17 (2.42%)	13 (4.98%)	1 (3.45%)
Clavien–Dindo IIIb, *n* (%)	43 (1.5%)	26 (1.15%)	11 (2.01%)	5 (8.20%)	1 (20.00%)	20 (1.06%)	12 (1.71%)	8 (3.07%)	3 (10.34%)
Clavien–Dindo IV, *n* (%)	31 (1.1%)	18 (0.80%)	9 (1.65%)	2 (3.28%)	2 (40.00%)	15 (0.80%)	8 (1.14%)	8 (3.07%)	0 (0.00%)
Mortality, *n* (%)	6 (0.2%)	3 (0.13%)	1 (0.18%)	2 (3.28%)	0 (0.00%)	1 (0.05%)	4 (0.57%)	1 (0.38%)	0 (0.00%)
eLOS, *n* (%)	35 (1.2%)	22 (0.97%)	9 (1.65%)	2 (3.28%)	2 (40.00%)	16 (0.85%)	8 (1.14%)	8 (3.07%)	3 (10.34%)
Nonhome discharge, *n* (%)	32 (1.1%)	20 (0.89%)	9 (1.65%)	2 (3.28%)	1 (20.00%)	13 (0.69%)	8 (1.14%)	10 (3.83%)	1 (3.45%)

Abbreviations: CHF, congestive heart failure; Dehisc, wound dehiscence; DSSI, deep incisional site infection; eLOS, extended length of stay; OSSI, organ/space surgical site infection; SSI, superficial incisional site infection.

^a^ Unknown/Native Hawaiian or Other Pacific Islander/American Indian or Alaska Native.

The overall postoperative mortality rate was low, while clinically significant morbidity remained notable. Rates of postoperative adverse events included CD II complications (Table S2), eLOS, and NHD, each increasing in frequency with higher frailty category by both mFI‐5 and RAI‐A.

### 3.2 Univariate Analysis

On unadjusted analysis, increasing RAI‐A score was significantly associated with postoperative mortality (*p* < 0.001), CD II–IV complications (*p* < 0.001), NHD (*p* < 0.001), and eLOS (*p* < 0.001). The mFI‐5 score demonstrated significant univariate associations with CD II (*p* = 0.005), CD IIIb (*p* = 0.009), eLOS (*p* < 0.001), and NHD (*p* = 0.006), but not with mortality (*p* = 0.072), DSSI (*p* = 0.638), nor OSSI (*p* = 0.635) (Table 2).

### 3.3 Multivariate Analysis

After adjustment for age, sex, BMI, smoking status, ASA class, and operative duration, the RAI‐A remained independently associated with multiple postoperative outcomes (Table 2). For mortality, RAI‐A scores of ≥ 31 (frail) were associated with significantly higher odds of death (OR 8.602, 95% CI 1.074–68.916; *p* = 0.043), whereas mFI‐5 did not independently predict mortality. The RAI‐A was also independently associated with CD II (OR 5.576, 95% CI 1.987–15.665; *p* < 0.001), CD IIIb (OR 4.264, 95% CI 1.419–12.811; *p* = 0.01), CD IV (OR 3.291, 95% CI 0.632–17.126; *p* = 0.157), NHD (OR 33.915, 95% CI 3.361–363.865; *p* < 0.001), and eLOS (OR 40.623, 95% CI 4.906–336.395; *p* < 0.001) (Table 3). By contrast, mFI‐5 predicted CD II (OR 13.68 for score of 4; 95% CI 1.399–133.754; *p* = 0.025) and CD IIIb (OR 10.956 for score of 4; 95% CI 2.563–46.836; *p* = 0.001), but it did not independently predict mortality or discharge disposition in adjusted models. RAI‐A significantly outperformed mFI‐5 in detecting patients at risk of postoperative SSI overall (RAI‐A: OR 7.545; 95% CI 2.367–24.048; *p* < 0.001), whereas mFI‐5 showed no significant association with superficial, deep, nor organ‐space infection.

**TABLE 3:** Multivariate analysis across frailty cohort and outcomes.

Complication	RAI‐A prefrail	RAI‐A frail	RAI‐A severely frail	mFI prefrail	mFI frail	mFI severely frail
*Mortality*	0.702 (0.068–7.226)	8.602 (1.074–68.916)*	—	2.601 (0.239–28.274)	2.018 (0.111–36.671)	—
CD I	2.271 (1.013–5.093)*	7.545 (2.367–24.048)**	—	0.781 (0.321–1.903)	0.845 (0.26–2.753)	2.068 (0.241–17.741)
CD II	2.001 (0.937–4.276)	5.576 (1.397–15.655)*	6.503 (0.294–143.945)	2.193 (0.928–5.185)	5.291 (1.932–14.49)*	13.68 (1.399–133.754)*
CD IIIb	1.412 (0.669–2.98)	4.264 (1.419–12.811)*	17.925 (1.641–195.967)*	1.48 (0.635–3.452)	2.584 (0.97–6.882)	10.956 (2.563–46.386)*
CD IV	1.892 (0.768–4.659)	3.291 (0.632–17.162)	41.472 (3.96–434.226)*	0.636 (0.221–1.831)	2.002 (0.736–5.444)	—
eLOS	1.567 (0.686–3.675)	4.623 (0.496–35.906)	40.623 (3.906–336.255)**	0.968 (0.331–2.831)	3.213 (1.127–9.154)	21.625 (4.618–101.325)*
*Nonhome discharge*	1.624 (0.701–3.765)	2.175 (0.453–10.45)	33.915 (3.121–368.265)*	1.384 (0.458–4.186)	5.695 (1.933–16.775)*	4.082 (0.767–28.232)
SSI	2.271 (1.013–5.093)*	7.545 (2.367–24.048)*	—	0.781 (0.321–1.903)	0.845 (0.26–2.753)	2.068 (0.241–17.741)
DSSI	2.141 (0.31–14.808)	—	—	1.576 (0.109–22.883)	2.96 (0.177–49.558)	—
OSSI	2.467 (0.669–9.091)	—	—	1.121 (0.27–4.652)	1.117 (0.192–6.511)	—
Wound dehiscence	2.19 (0.263–18.23)	—	—	5.287 (0.349–80.015)	5.663 (0.254–126.332)	—

Abbreviations: CD, Clavien–Dindo; DSSI, deep surgical site infection; eLOS, extended length of stay; OSSI, organ space/site infection; SSI, surgical site infection.

* *p* < 0.05.

** *p* < 0.001.

### 3.4 ROC Curve Analysis

**FIGURE 1:**
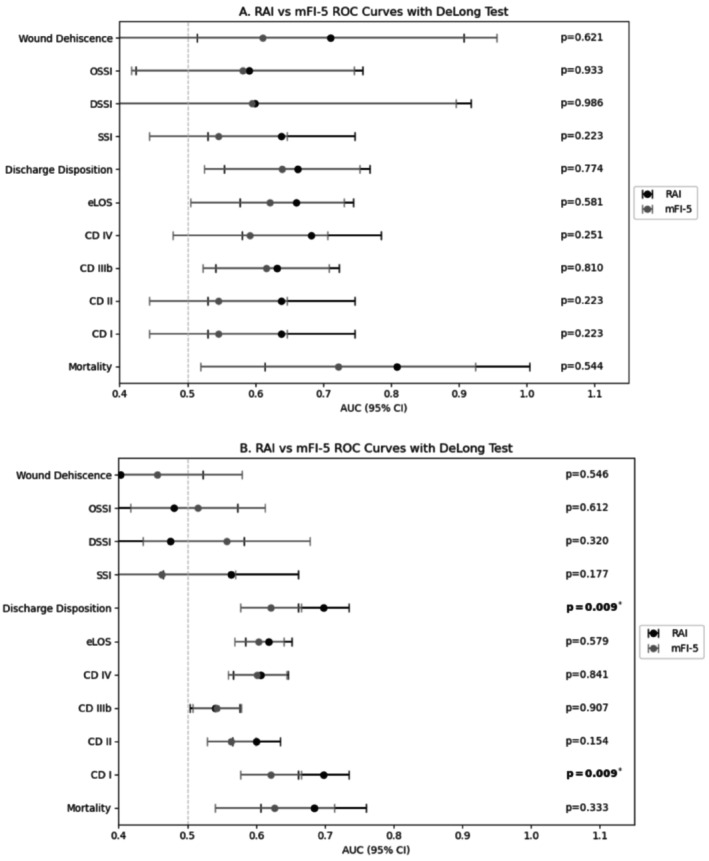
DeLong test comparing ROC C‐statistics across cohorts. (A) Overall otology cohort. (B) Otologic skull base cohort. *p < 0.05 . CD, Clavien–Dindo; DSSI, deep surgical site infection; eLOS, extended length of stay; OSSI, organ‐space/site infection; SSI, surgical site infection.

Receiver operating characteristic analysis demonstrated numerically higher AUCs for RAI‐A compared with mFI‐5 for multiple important outcomes (Table 3, Figure 1). The RAI‐A had higher AUC values for mortality (AUC 0.809 vs. 0.722), CD II complications (0.682 vs. 0.592), eLOS (0.660 vs. 0.621), discharge disposition (0.662 vs. 0.639), and SSI (0.638 vs. 0.546). Age alone performed worse than both frailty indices across all outcomes except mortality, confirming the clinical value added by frailty measurement (Table 4, Figure 1).

**TABLE 4:** Receiver operating characteristic analysis.

Frailty metric	Otology cohort AUC (95% CI)	Skull base cohort AUC (95% CI)
RAI‐A mortality	0.809 (0.614–1.004)	0.684 (0.607–0.760)
mFI mortality	0.722 (0.519–0.924)	0.627 (0.540–0.714)
Age mortality	0.759 (0.522–0.996)	0.636 (0.553–0.719)
RAI‐A eLOS	0.660 (0.577–0.744)	0.698 (0.661–0.735)
mFI eLOS	0.621 (0.512–0.731)	0.621 (0.577–0.665)
Age eLOS	0.637 (0.555–0.719)	0.687 (0.645–0.729)
RAI‐A discharge disposition	0.662 (0.554–0.769)	0.600 (0.566–0.635)
mFI discharge disposition	0.639 (0.525–0.754)	0.564 (0.529–0.599)
Age discharge disposition	0.633 (0.526–0.739)	0.568 (0.532–0.603)
RAI‐A CD I	0.638 (0.53–0.746)	0.540 (0.504–0.576)
mFI CD I	0.546 (0.444–0.647)	0.543 (0.508–0.578)
Age CD I	0.639 (0.538–0.741)	0.527 (0.490–0.563)
RAI‐A CD II	0.638 (0.53–0.746)	0.607 (0.567–0.647)
mFI CD II	0.546 (0.444–0.647)	0.601 (0.559–0.644)
Age CD II	0.639 (0.538–0.741)	0.570 (0.527–0.612)
RAI‐A CD IIIb	0.632 (0.541–0.723)	0.618 (0.585–0.652)
mFI CD IIIb	0.616 (0.522–0.709)	0.604 (0.569–0.640)
Age CD IIIb	0.618 (0.533–0.703)	0.589 (0.554–0.624)
RAI‐A CD IV	0.682 (0.584–0.780)	0.698 (0.661–0.735)
mFI CD IV	0.592 (0.478–0.706)	0.621 (0.577–0.665)
Age CD IV	0.641 (0.549–0.734)	0.687 (0.645–0.729)
RAI‐A DSSI	0.599 (0.279–0.918)	0.563 (0.465–0.661)
mFI DSSI	0.595 (0.294–0.896)	0.463 (0.355–0.570)
Age DSSI	0.624 (0.33–0.917)	0.539 (0.444–0.634)
RAI‐A SSI	0.638 (0.53–0.746)	0.475 (0.369–0.582)
mFI SSI	0.546 (0.444–0.647)	0.557 (0.435–0.678)
Age SSI	0.639 (0.538–0.741)	0.504 (0.363–0.644)
RAI‐A OSSI	0.591 (0.424–0.758)	0.480 (0.386–0.573)
mFI OSSI	0.581 (0.417–0.745)	0.515 (0.417–0.613)
Age OSSI	0.624 (0.433–0.814)	0.486 (0.393–0.579)
RAI‐A wound dehiscence	0.711 (0.514–0.907)	0.403 (0.283–0.523)
mFI wound dehiscence	0.611 (0.267–0.956)	0.456 (0.334–0.579)
Age wound dehiscence	0.696 (0.457–0.935)	0.391 (0.274–0.507)

Abbreviations: CD, Clavien–Dindo; DSSI, deep surgical site infection; eLOS, extended length of stay; OSSI, organ space/site infection; SSI, surgical site infection.

### 3.5 Lateral Skull Base Subgroup Analysis

A predefined subgroup analysis was performed for patients undergoing lateral skull base surgery (*n* = 56). Compared with the overall cohort, lateral skull base patients demonstrated a higher prevalence of frailty by both indices and experienced higher postoperative morbidity. On multivariate modeling, increasing RAI‐A score remained significantly associated with several key postoperative outcomes, including CD II complications (OR 3.56, 95% CI 1.36–9.31; *p* = 0.01), mortality (OR 14.47, 95% CI 3.02–69.31; *p* < 0.001), and NHD (OR 18.20, 95% CI 6.35–52.17; *p* < 0.001). In contrast, mFI‐5 did not demonstrate statistically significant associations with these outcomes in this high‐risk subgroup. ROC curve analysis supported these findings, demonstrating superior discriminatory capability of the RAI‐A for mortality (RAI‐A AUC 0.684 vs. mFI‐5 AUC 0.627), eLOS (RAI‐A AUC 0.698 vs. mFI‐5 AUC 0.621), and discharge disposition (RAI‐A AUC 0.600 vs. mFI‐5 AUC 0.564) (Table 5).

**TABLE 5:** Otologic skull base cohort multivariate analysis.

Complication	RAI‐A prefrail	RAI‐A frail	RAI‐A severely frail	mFI prefrail	mFI frail	mFI severely frail
*Mortality*	1.023 (0.44–2.376)	4.219 (1.129–15.796)*	14.466 (3.019–69.309)**	1.517 (0.692–3.323)	1.283 (0.473–3.478)	4.104 (0.814–20.691)
CD I	0.684 (0.259–1.811)	2.003 (0.56–7.157)	8.798 (1.044–74.166)*	0.58 (0.233–1.442)	0.349 (0.075–1.612)	1.753 (0.207–14.814)
CD II	2.142 (1.597–2.874)**	3.785 (2.373–6.037)**	10.238 (3.229–32.464)**	1.37 (0.999–1.88)	2.794 (1.877–4.161)**	8.425 (3.633–19.539)**
CD IIIb	1.449 (0.956–2.199)	1.44 (0.492–4.206)	3.063 (0.294–31.914)	1.175 (0.852–1.619)	1.449 (0.937–2.239)	1.958 (0.705–5.439)
CD IV	2.514 (1.456–4.352)**	3.556 (1.356–9.324)*	—	1.492 (1.031–2.16)*	2.344 (1.554–3.812)**	—
eLOS	2.094 (1.558–2.814)**	2.527 (1.525–4.189)**	3.948 (1.081–14.423)*	1.696 (1.229–2.341)**	2.711 (1.811–4.059)**	3.551 (1.43–8.815)**
*Discharge disposition*	3.865 (2.712–5.508)**	2.977 (1.578–5.617)**	18.196 (6.346–52.712)**	1.395 (0.942–2.064)	1.837 (1.123–3.003)*	5.103 (1.429–12.711)*
SSI	0.684 (0.259–1.811)	2.003 (0.56–7.157)	8.798 (1.044–74.166)*	0.58 (0.233–1.442)	0.349 (0.075–1.612)	1.753 (0.207–14.814)
DSSI	1.548 (0.498–4.81)	—	—	1.392 (0.427–4.542)	1.318 (0.247–7.023)	—
OSSI	0.644 (0.269–1.542)	0.457 (0.06–3.459)	—	1.149 (0.495–2.668)	1.546 (0.512–4.667)	—
Wound dehiscence	0.892 (0.328–2.425)	—	—	0.954 (0.329–2.763)	0.875 (0.177–4.329)	—

Abbreviations: CD, Clavien–Dindo; DSSI, deep surgical site infection; eLOS, extended length of stay; OSSI, organ space/site infection; SSI, surgical site infection.

* *p* < 0.05.

** *p* < 0.001.

## 4 Discussion

In this large retrospective cohort study of 2862 otologic and neurotologic surgery patients, frailty was strongly associated with postoperative morbidity and discharge disposition. Increasing RAI‐A score was independently associated with greater than eightfold higher odds of 30‐day mortality, 5.6‐fold higher odds of CD II complications, 41‐fold higher odds of eLOS, and 33‐fold higher odds of NHD. While the RAI‐A consistently demonstrated higher AUC values than mFI‐5 across clinically important outcomes, DeLong testing did not demonstrate statistically significant differences in the overall otologic cohort (all *p* > 0.05). In the lateral skull base subgroup, however, RAI‐A demonstrated significantly greater discriminatory accuracy for CD I complications and NHD compared with mFI‐5 (both *p* = 0.009), indicating potential added value in high‐complexity procedures (Figure 1). While several observed differences did not reach statistical significance, even modest improvements in discrimination may yield clinically meaningful gains in perioperative planning, discharge forecasting, and patient counseling. These findings suggest that the RAI‐A more accurately captures physiologic vulnerability and postoperative recovery potential in routine and complex otologic procedures.

### 4.1 Existing Literature

Although frailty research in otolaryngology and related neurosurgical domains has expanded rapidly, there remains no consensus on the optimal instrument for preoperative risk stratification, especially within otology. Much of the early literature in these domains emphasized the mFI‐5 and mFI‐11, which are associated with higher rates of postoperative complications and resource use; however, most studies benchmarked these indices against age alone, a limited comparator for surgical risk [12, 16, 17, 18, 19]. Moreover, fidelity to the frailty construct requires assessment across multiple domains—often on the order of 10–30 variables—rather than a short comorbidity list [20]. The mFI‐5 is effectively a four‐item comorbidity scale (diabetes, COPD, CHF, hypertension) plus a single binary functional status item; its equal weighting of disparate factors can misrepresent physiologic reserve (e.g., equating hypertension with functional dependence). In contrast, the RAI‐A was explicitly designed to measure frailty, incorporates broader domains (age, activities of daily living, living situation, weight loss, dyspnea, and comorbidities) with graded contributions, and has been validated retrospectively and prospectively across multiple surgical specialties [21, 22, 23, 24].

### 4.2 RAI‐A vs. mFI‐5

Consistent with its conceptual foundation, this study's findings demonstrate the superiority of the RAI‐A in identifying physiologic vulnerability and predicting clinically meaningful outcomes. Multivariable modeling further refined these observations; after adjusting for conventional risk factors, the RAI‐A remained an independent predictor of mortality, CD II and IIIb complications, eLOS, and failure to return home, whereas the mFI‐5 did not.

ROC curve performance provided additional insight. Although the RAI‐A consistently demonstrated numerically greater discriminatory accuracy than mFI‐5 in the overall cohort, these differences did not reach statistical significance. This likely reflects the low event rates and high physiologic resilience in routine otologic surgery, resulting in limited statistical power to detect modest AUC differences. Importantly, however, real‐world clinical utility should not be judged solely by statistical significance—the observed AUC advantages may still translate into meaningful improvements in patient selection, discharge planning, and resource allocation.

In contrast, statistically significant performance differences emerged in the lateral skull base subgroup, where the RAI‐A outperformed mFI‐5 in predicting CD I complications and NHD. Despite the limited sample size, the RAI‐A remained independently predictive of CD II complications, mortality, and NHD, whereas the mFI‐5 failed to demonstrate independent associations with these outcomes. Given the intrinsic complexity and higher acuity of lateral skull base procedures, these findings suggest that the RAI‐A is particularly effective at capturing physiologic reserve in patients undergoing demanding neurotologic operations—a setting where frailty assessment is most critical for optimizing surgical decision‐making, expectation management, and postoperative planning. These patterns align with findings from neurosurgery, orthopedic surgery, and general surgery, where the RAI‐A has consistently outperformed the mFI‐5 as a risk‐stratification tool for postoperative morbidity and mortality, particularly in high‐acuity populations [21, 25, 26, 27, 28, 29, 30].

### 4.3 Clinical Utility

Incorporating RAI‐based frailty screening into routine preoperative evaluation can directly inform perioperative management in otology and neurotology. Notably, the principal advantage of the RAI‐A lies in its multidimensional structure and validated clinical interpretability, whereas its primary limitation is a slightly greater data dependency compared with simpler indices such as the mFI‐5. However, this tradeoff seems marginal, especially as most of the RAI‐A variables are routinely documented in patient charts. In the preoperative phase, elevated RAI‐A can trigger targeted optimization (nutrition support, anemia management, glycemic control, medication reconciliation), anesthesia and airway planning, and early geriatric/medicine co‐management when appropriate [31, 32]. For operative planning, high‐frailty patients can be scheduled with senior teams, longer room time, and proactive ICU/PACU bed allocation for lateral skull base cases [29, 33, 34]. Postoperatively, RAI‐A can guide enhanced recovery pathways tailored to frailty (early mobilization, multimodal analgesia to limit delirium risk, vestibular rehabilitation, swallow assessment when indicated, aggressive pulmonary hygiene, and SSI‐prevention bundles), along with early PT/OT, social work involvement, and arrangement of home services or facility placement to reduce NHD failure and readmissions [31, 32, 33, 35, 36, 37, 38, 39, 40]. At the system level, embedding RAI‐A in preoperative clinics facilitates risk‐adjusted counseling, shared decision‐making, and more accurate forecasting of resource needs. Evidently, integrating an RAI‐based frailty screen into otologic and lateral skull base perioperative pathways offers actionable risk stratification that can inform optimization, resource planning, and shared decision‐making.

## 5 Limitations

A few limitations should be acknowledged and are largely attributable to the retrospective design and use of an administrative dataset. Although NSQIP maintains rigorous data quality standards through routine auditing, its structure restricts outcome capture to the first 30 postoperative days and lacks procedure‐specific nuance such as disease severity and anatomic complexity. Because only patients who were deemed appropriate surgical candidates are included, selection bias is possible, as the most severely frail individuals may not have been offered surgery. Several outcomes were rare, resulting in wide confidence intervals; these findings should be interpreted as directionally informative rather than as precise point estimates. Additionally, although certain otologic CPT codes may also be performed in outpatient clinic settings, the NSQIP includes both inpatient and outpatient surgical encounters abstracted by trained reviewers using standardized methods. However, sensitivity analysis excluding these lower‐acuity procedures yielded consistent results, suggesting that their inclusion did not meaningfully influence the comparative performance of frailty indices. Frailty was assessed using the retrospective RAI‐A rather than the prospective clinical RAI‐C, which may introduce measurement variability; however, both versions have been extensively validated in surgical populations. Additionally, although this study focused on discrimination, future work incorporating calibration metrics such as Brier scores or calibration plots may further clarify the clinical performance of frailty‐based models. Finally, the lateral skull base subgroup contained relatively few adverse events, which limits precision of estimates within this high‐risk cohort. Despite these limitations, the large national sample and standardized outcomes support the robustness and generalizability of our findings.

## 6 Conclusion

The RAI provides a more comprehensive indicator of physiologic reserve than the mFI‐5 and was a stronger predictor of morbidity, mortality, and discharge disposition following otologic and lateral skull base surgery. The RAI‐A demonstrated improved performance in high‐complexity skull base procedures compared with routine otologic surgery. Integrating frailty screening using the RAI‐A may enhance perioperative counseling, optimize resource planning, and improve postoperative care. Prospective studies are needed to further define the role of RAI‐based frailty assessment across larger and more diverse otologic surgical cohorts.

## Funding

The authors have nothing to report.

## Conflicts of Interest

The authors declare no conflicts of interest.

## Supporting information


**Table S1:** Otology CPT codes.
**Table S2:** Clavien–Dindo classification system.

## Data Availability

The data that support the findings of this study are openly available in ACS National Surgical Quality Improvement Program at https://www.facs.org/quality‐programs/data‐and‐registries/acs‐nsqip/.
