# Supplementary material for: Otologic Surgery Risk Prediction: Risk Analysis Index‐Administrative Versus Modified Frailty Index‐5
Source: Laryngoscope. 2026 Apr 3;136(9):3955–65. doi: 10.1002/lary.70539 (PMC13460496; doi:10.1002/lary.70539)
Supplement: Supplementary file 1 [file LARY-136-3955-s001.docx]

**Supplemental Table 1**: Otology CPT Codes

| **CPT Code** | **Official Descriptor** | **Typical Use Case** |
| --- | --- | --- |
| *External Ear (CPT 69000-69399)* | | |
| Incision/Drainage | | |
| 69000 | Drain external auditory canal abscess | Drain EAC abscess |
| 69005 | Drain external ear hematoma | Auricular hematoma evacuation |
| 69020 | Incision of external ear | I&D auricle abscess/cyst |
| Excision | | |
| 69100 | Excision external ear lesion simple | Skin lesion excision – localized |
| 69105 | Excision external ear lesion w/ margin | Wider skin lesion excision |
| 69110 | Excision external ear lesion complex | Complex benign/malignant lesions |
| 69120 | Partial auriculectomy | Cancer requiring partial ear removal |
| 69140 | Complete auriculectomy | Total ear removal for malignancy |
| 69145 | Reconstruction external ear | Auricular reconstruction |
| 69150 | Revision auricular reconstruction | Secondary reconstruction |
| 69155 | External auditory canal mass excision | Excision of EAC neoplasm |
| Foreign Body/Wax Removal | | |
| 69200 | Foreign body removal external ear | Pediatric FB removal |
| 69205 | Removal FB w/ anesthesia | Complex FB removal |
| 69209 | Cerumen removal lavage/irrigation | Wax removal without instruments |
| 69210 | Cerumen removal manual | Manual wax disimpaction |
| 69220 | Aural polyp removal simple | EAC polyp |
| 69222 | Aural polyp removal extensive | Larger polyp/infection |
| Reconstruction | | |
| 69300 | Canalplasty | Repair congenital/acquired stenosis |
| 69310 | Split-thickness graft external ear | Reconstruction post excision/trauma |
| 69320 | Reconstruction EAC | Surgical repair of stenosis |
| Miscellaneous | | |
| 69399 | Unlisted external ear procedure | Rare/excluded procedures |
| *Middle Ear / Mastoid (CPT 69400–69700)* | | |
| Incision Procedures | | |
| 69420 | Eustachian tube dilation | Balloon dilation for ET dysfunction |
| 69433 | PE tube insertion GA | Tube placement under anesthesia |
| 69436 | Tympanostomy general anesthesia | Myringotomy with tubes |
| 69440 | Myringotomy without tube | Simple ME drainage |
| 69450 | Myringotomy with tube | Tube placement adult |
| Excision Procedures | | |
| 69501 | Excise ET lesion | ET tumor removal |
| 69502 | Middle ear tumor excision | Cholesteatoma/mass excision |
| 69530 | Tympanotomy with excision | Tumor/mass removal |
| 69540 | Aural polyp excision aggressive | Polyp from chronic otitis |
| 69550–69554 | Middle ear glomus tumor excision | Excision paraganglioma |
| Repair Procedures | | |
| 69601–69606 | Repair tympanic membrane | TM perforation repair |
| 69610 | Myringoplasty | Limited perforation closure |
| 69620 | Myringoplasty revision | Repeat repair |
| 69631 | Tympanoplasty no OCR | TM+ME disease without OCR |
| 69632 | Tympanoplasty with OCR | Ossicular chain reconstruction |
| 69633–69646 | Complex tympanoplasty ± mastoidectomy | Chronic otitis/cholesteatoma |
| 69676 | Revision mastoidectomy | Recurrence/cavity issues |
| Other Middle Ear Procedures | | |
| 69700 | Middle ear exploration | Evaluate conductive HL |
| 69705 | Osseointegrated implant placement initial | Bone-anchored hearing implant |
| 69706 | Osseointegrated implant revision | Abutment revision |
| 69710 | Implant Osseointegrated with mastoid drilling | BAHA/HANS system |
| Implants (Overlap with Inner ear) | | |
| 69711–69718 | Advanced Osseointegrated implant systems | Transcutaneous implant options |
| 69930 | Cochlear implant | Bilateral profound SNHL |
| *Inner Ear / Facial Nerve (CPT 69720-69979)* | | |
| Facial Nerve / Vestibular Procedures | | |
| 69949 | Auditory brainstem implant | NF2 or non-implantable cochlea |
| 69720 | Facial nerve decompression | Bell’s palsy/trauma |
| 69725 | Facial nerve repair/graft | Reconstruction after injury |
| 69740–69779 | Vestibular/labyrinth procedures | Labyrinthectomy/vestibular nerve |
| Labyrinth Procedures | | |
| 69905 | Labyrinthectomy | Ménière’s disease refractory |
| 69910 | Endolymphatic sac decompression | Ménière’s decompression |
| 69915 | Cochleostomy | Access for electrode/inner ear drainage |
| 69920 | Drain petrous apex | Cholesterol granuloma drainage |
| 69931–69979 | Inner ear/facial nerve advanced procedures | Rare skull base/inner ear |
| *Skull Base (CPT 61580-61616)* | | |
| 61580–61616 | Skull base approaches temporal bone | VS, glomus tumors, petrosectomy |

**Supplemental Table 2:** Clavien-Dindo Classification System^40^

| **Grade** | **Definition** |
| --- | --- |
| Grade I | Any deviation from the normal postoperative course without the need for pharmacological, surgical, endoscopic, or radiological interventions. Allowed therapeutic measures include antiemetics, antipyretics, analgesics, diuretics, electrolytes, and physiotherapy. Includes wound infections treated at the bedside. |
| Grade II | Complications requiring pharmacologic treatment beyond Grade I medications. Includes blood transfusion and total parenteral nutrition |
| Grade III | Complications requiring surgical, endoscopic, or radiological intervention |
| *Grade IIIa* | Intervention without general anesthesia |
| *Grade IIIb* | Intervention under general anesthesia |
| Grade IV | Life-threatening complications requiring ICU or management of organ dysfunction |
| *Grade IVa* | Single organ dysfunction |
| *Grade IVb* | Multi-organ dysfunction |
| Grade V | Patient Death |
| Suffix “d” | Indicates complications causing disability at discharge requiring follow‑up (e.g. “IIIb‑d”). |
